# Coacervates as bio-inspired intermediates for materials processing

**DOI:** 10.1039/d6cc03055f

**Published:** 2026-08-17

**Authors:** Chantal Graafsma, Malak Rafieq Jaber, Guillermo Monreal Santiago, Marleen Kamperman

**Affiliations:** a Zernike Institute for Advanced Materials, University of Groningen, Nijenborgh 3 9747 AG Groningen The Netherlands marleen.kamperman@rug.nl; b UMR7140 – Chimie de la Matière Complexe, CNRS, Université de Strasbourg, 4 Rue Blaise Pascal Strasbourg 67081 France

## Abstract

Nature routinely fabricates fibers and composites with combinations of toughness, strength, gradients, and multifunctionality that remain challenging for synthetic materials. A growing body of evidence indicates that many of these materials are not processed from dilute solutions or polymer melts, but instead *via* concentrated, liquid-like macromolecular phases known as coacervates. In this Feature Article, we review how coacervation enables aqueous, highly controlled processing of fibers and related materials. We place recent work from our groups in the broader context of natural and synthetic coacervate research, discuss established and emerging processing strategies, and outline a perspective in which gradual, stimulus-driven changes in solubility and intermolecular interactions are exploited to guide structure formation and material properties.

## Abstract 2

Controlled processing in natural coacervate systems provides inspiration for synthetic materials design.

## Graphical abstract

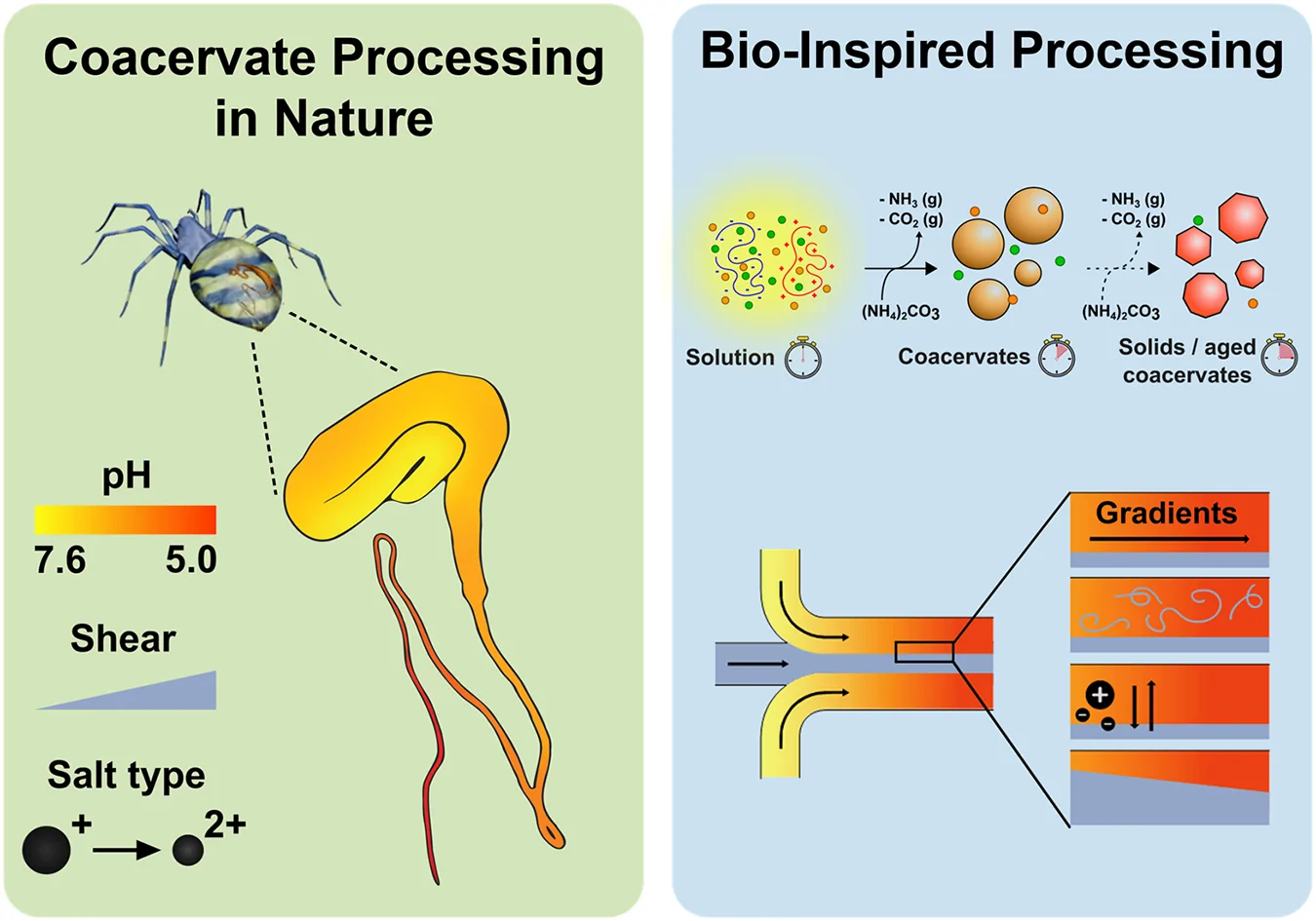

## 1. Introduction: processing as the missing link in functional materials

Polymer processing plays a decisive role in determining the structure and performance of materials, yet conventional processing routes often provide only limited control. High-performance synthetic fibers typically rely on organic solvents and extreme processing conditions to induce chain alignment and mechanical strength.^1^ In contrast, biological systems routinely fabricate fibers and composites under mild, aqueous conditions while achieving exceptional combinations of toughness, stiffness, extensibility, and functional gradients.^2^ Spider silks,^3–5^ squid beaks,^6,7^ velvet worm slime,^8,9^ and underwater adhesives^10–12^ are illustrative examples. The remarkable performance of these materials cannot be attributed to molecular composition alone. Instead, it emerges from tightly regulated processing pathways that guide structure formation as the material is being fabricated.

A growing body of evidence indicates that many biological materials are processed from coacervates—dense, phase-separated polymer fluids—rather than from dilute solutions or solid precursors.^13^ These liquid-like intermediates permit flow, deformation, and reorganization while maintaining high macromolecular concentrations and strong intermolecular interactions. Processing in such a state allows biological systems to continuously steer molecular alignment, supramolecular assembly, and hydration during shaping, before the structure is ultimately fixed.

Recognizing coacervates as processing intermediates shifts the focus from material composition to dynamic pathways of assembly and solidification. This perspective suggests that controlling how interactions evolve during processing is central to achieving the extraordinary properties of natural fibers and provides inspiration for synthetic materials design.

## 2. Coacervation in natural materials

Coacervation refers to an entropy-driven, associative liquid–liquid phase separation in which macromolecules condense into a dense, polymer-rich phase that coexists with a dilute aqueous phase.^14^ Coacervates are typically stabilized by electrostatic interactions, often complemented by hydrogen bonding, hydrophobic interactions, or π–π interactions.^15^ Containing 60–90% water, coacervates behave as viscoelastic liquids, characterized by low interfacial tension, high macromolecular concentration, and pronounced responsiveness to environmental conditions. The process of coacervation is governed by an intricate interplay of thermodynamic driving forces, such as the enthalpy-driven attraction between polymer groups and the entropically favorable release of counterions and/or water molecules. While outside the scope of this work, interested readers may consult dedicated reviews for an in-dept discussion of this topic.^16,17^

**Fig. 1:**
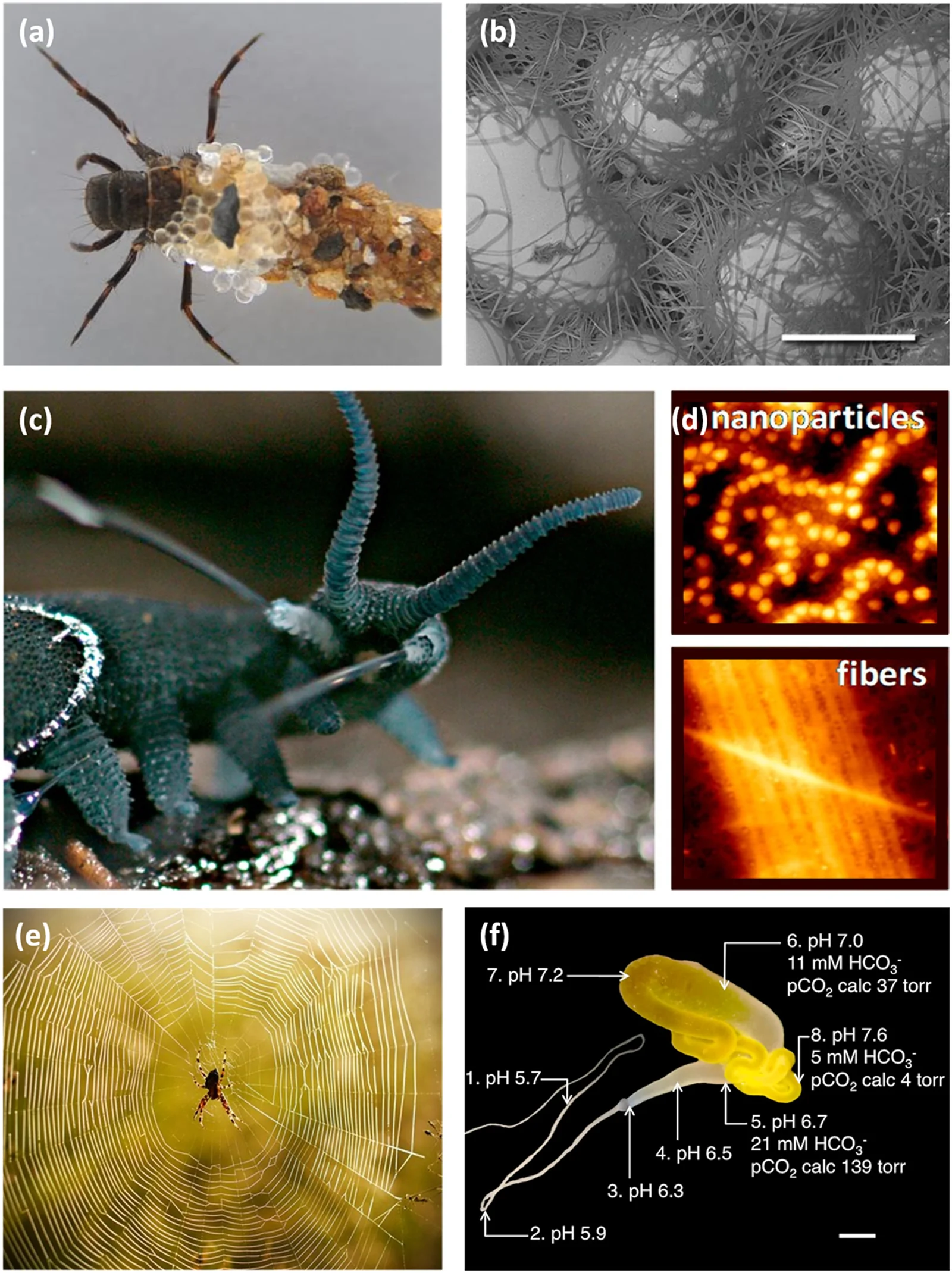
(a) and (b) Caddisfly larvae spin silk to build tubular structures (scale bar 250 µm). Reproduced with permission from ref. 23. Copyright© 2010 American Chemical Society. (c) and (d) Velvet worms extrude adhesive slime that is comprised of nanoglobules that transition to fibers. Reproduced with permission from ref. 9. Copyright© 2018 American Chemical Society. (e) Spiders spin silk fibers (Matt Crane, Getty Images). (f) Photograph of a spider major ampullate gland showing gradients along the duct (scale bar 1 mm). Reproduced from ref. 18, under the terms of the CC-BY 4.0 license. Copyright© 2014 Andersson *et al*.

Over the past decade, coacervates have been increasingly recognized as key intermediates in the processing of natural functional materials (Fig. 1). Spider silk is one of the most intensively studied examples.^2,4,5^ Silk proteins are stored in the major ampullate gland at extremely high concentrations in a fluid, condensed state.^3^ Upon spinning, the material is exposed to a tightly regulated sequence of changes in pH, ionic composition, confinement, and shear.^18^ These gradual changes induce conformational rearrangements and molecular alignment, leading to the formation of β-sheet-rich nanocrystalline domains that ultimately determine the mechanical performance of the fiber.^19,20^ The ability to process silk proteins as a dense fluid prior to solidification is widely believed to be essential for achieving the exceptional toughness of spider silk.^21^ Similar principles are observed in other fiber-forming systems. Velvet worms deploy a protein-based slime to capture prey, ejecting it as a fluid that rapidly transforms into viscoelastic fibers under extensional flow. Recent studies suggest that the slime consists of a dispersion of nanoglobular entities with coacervate-like characteristics, which reorganize and fuse during deformation.^8^ In this case, mechanical forces play a central role in triggering the transition from a liquid processing state to a solid or semi-solid fiber network.^9^ Mussels secrete byssal thread precursors as fluid coacervates that can readily wet and infiltrate substrates.^10^ Upon exposure to seawater, changes in pH, followed by biologically regulated curing reactions, lead to hardening and the development of tough, self-healing adhesive threads.^22^ Caddisfly larvae employ a related strategy, spinning underwater adhesive fibers from protein-rich secretions that reorganize, align, and gradually dehydrate after extrusion.^23,24^

Beyond macroscopic fibers and adhesives, coacervation has also been implicated in the formation of functional gradients and composites in nature. In squid beaks, for example, a pronounced gradient in stiffness and hardness is established through the infiltration of a protein-rich coacervate into a chitin-based scaffold, followed by spatially controlled crosslinking and structural rearrangement.^7,25,26^

Microscopically, stress granules formed in the cytosol *via* liquid–liquid phase separation present another example of coacervation leading to hierarchal organization in nature.^27^ Driven by RNA–protein interactions, these condensates adopt dynamic core–shell structures in response to cellular physiochemical conditions.^28^

Taken together, these examples reveal a common processing logic. Coacervation enables organisms to concentrate macromolecules, maintain processability, and respond sensitively to changes in chemical and mechanical cues. Gradual variations in solubility, hydration, and interaction strength guide supramolecular assembly and solidification in space and time, allowing complex hierarchical structures to emerge under mild, aqueous conditions. Recognizing this shared physical framework provides a powerful basis for translating biological processing strategies into synthetic material systems. This has motivated the development of synthetic and bio-derived coacervate systems, most prominently complex coacervates formed by oppositely charged polyelectrolytes.^11,29^

**Fig. 2:**
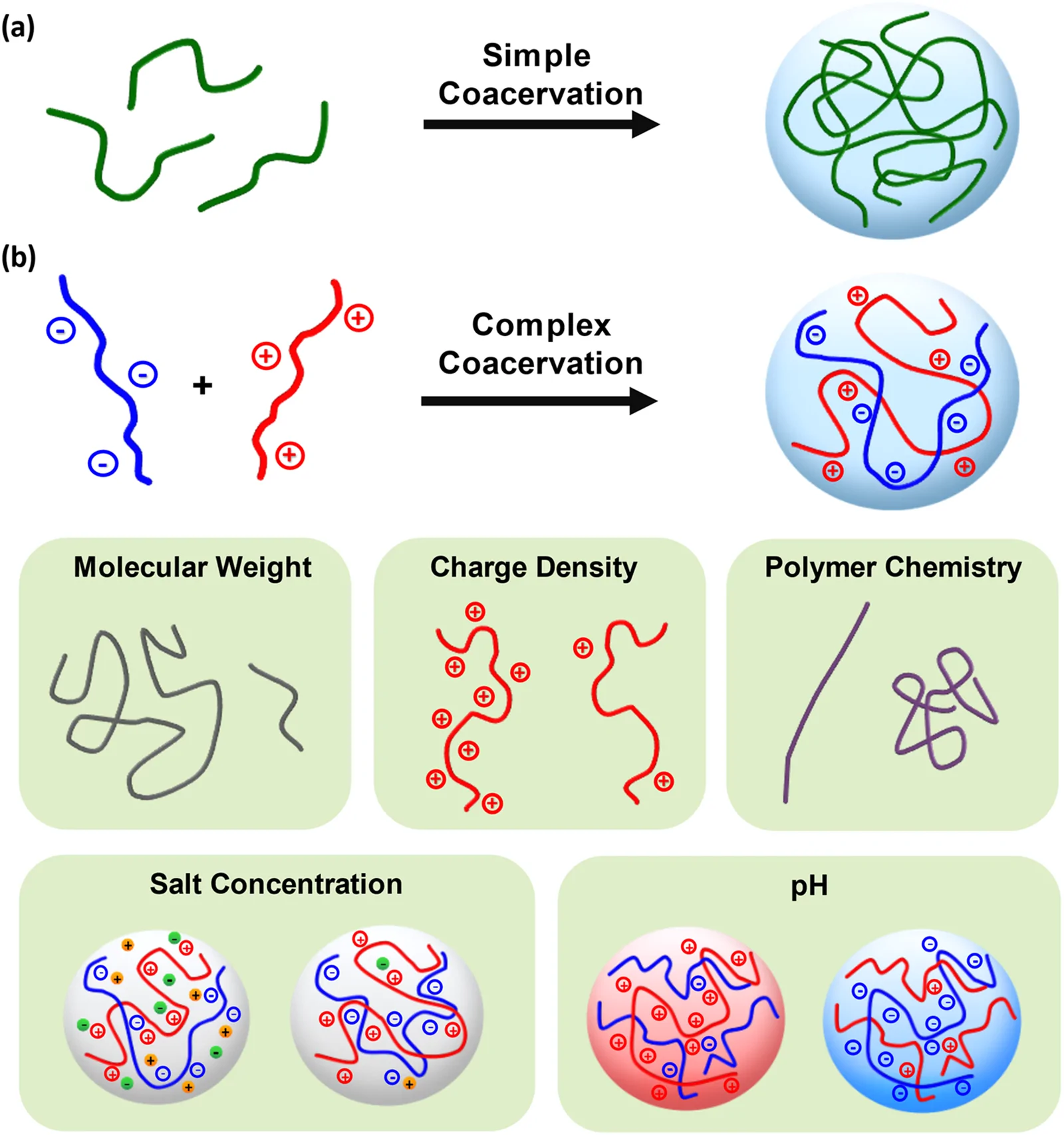
Schematic illustration of (a) simple and (b) complex coacervate formation and the key parameters that govern its phase behavior, interaction strength, rheological properties, and resulting solidification.

Complex coacervates offer a versatile and tunable platform. Their phase behavior and rheological properties can be adjusted through molecular weight, charge density, polymer chemistry, and environmental parameters such as salt concentration and pH (Fig. 2).^30–33^ Importantly, these systems retain the dynamic and responsive nature of biological coacervates, while offering scalability and chemical flexibility. As such, they provide an ideal testbed to explore bio-inspired processing concepts.

## 3. Environmental switching as a processing strategy for coacervates

The recognition of coacervates as liquid processing intermediates has enabled a wide range of fabrication strategies for fibers,^34,35^ films,^36^ membranes,^37^ microcapsules,^38^ and three-dimensional structures.^39^ A defining feature of these approaches is that shaping is performed while the material resides in a dense, yet fluid, state, after which solidification is triggered by controlled changes in environmental conditions. In our work, we have focused on exploiting this processing window through environmental switching, using simple changes in ionic strength, pH, or temperature to reversibly tune intermolecular interactions and thereby control processability and solidification.

Environmental switching builds on the intrinsic sensitivity of coacervates to their surroundings. Because coacervates are held together by non-covalent interactions, modest changes in environmental parameters can lead to pronounced changes in rheological response. This provides a powerful handle to switch coacervates between a highly processable, fluid state and a mechanically robust, solid-like state without the need for covalent crosslinking.^40,41^ We have used this principle as a unifying framework for the development of bioinspired adhesives, printable coacervate inks, and responsive soft materials.

### 3.1. Salt-mediated switching: plasticization and solidification

**Fig. 3:**
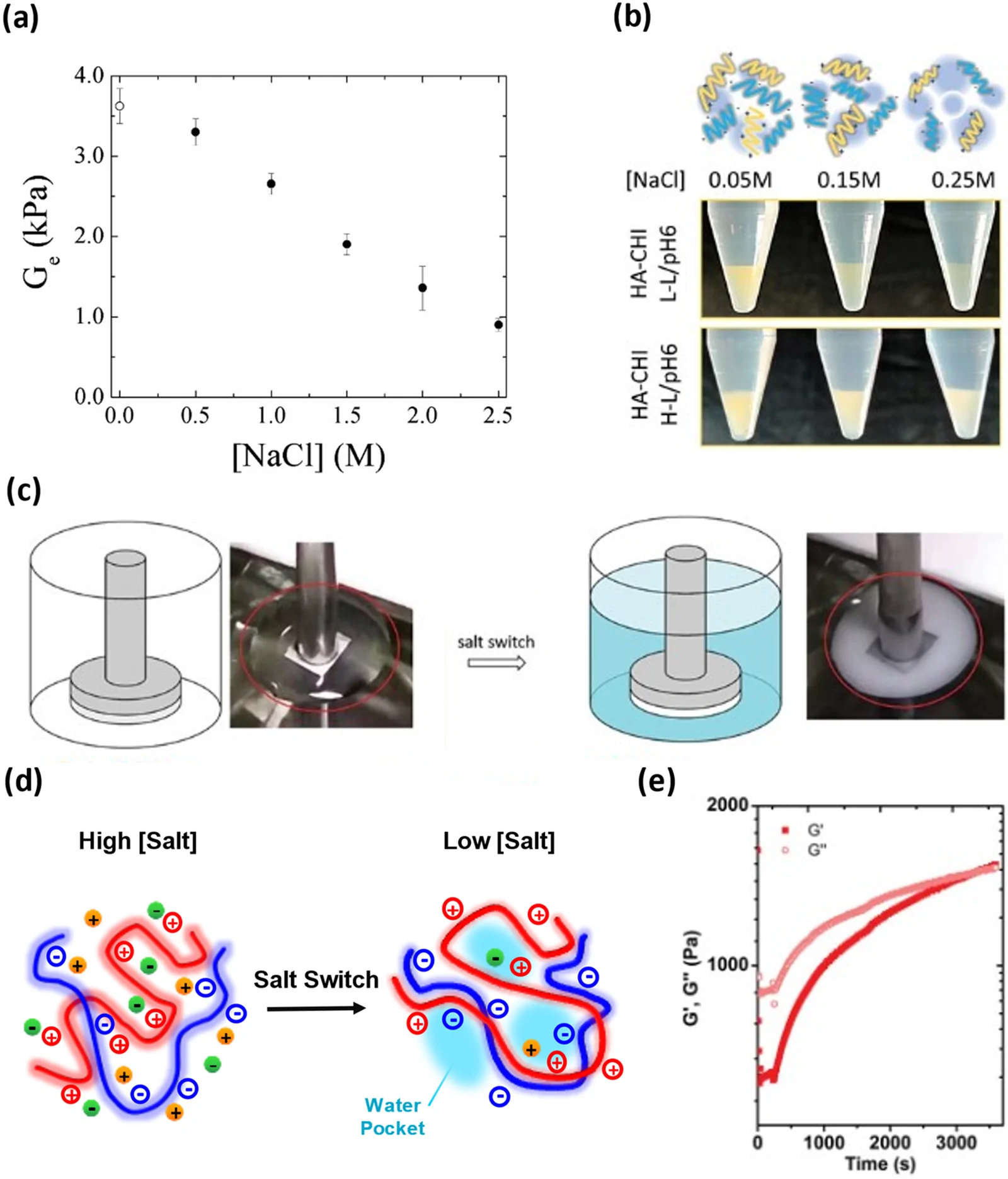
Salt-mediated switching of complex coacervates. (a) Equilibrium shear modulus of a PSS/PDADMA complex coacervate as a function of NaCl concentration. Reproduced from ref. 40, under the terms the ACS AuthorChoice license. Copyright© 2009 American Chemical Society. (b) Pictures of centrifuged complex coacervates with NaCl concentrations of 0.05, 0.15, and 0.25 M. H and L refers to high and low molecular weight of the hyaluronic acid and chitosan components. Reproduced from ref. 39, under the terms of the CC-BY-NC 4.0 license. Copyright© 2023 Khoonkari *et al*. Advanced Materials published by Wiley-VCH GmbH. (c) Schematic and pictures of solidification of a complex coacervate adhesive: the material transitions from a transparent viscous liquid to a white soft gel. Reproduced from ref. 44, under the terms of the CC-BY-NC 4.0 license. Copyright© 2019 Dompé *et al*. Published by Wiley-VCH Verlag GmbH & Co. KGaA, Weinheim. (d) Schematic representation of salt diffusion from a complex coacervate. Before the salt switch (left), counterions are largely associated with the polyelectrolyte chains and the adhesive behaves as a viscous liquid. After the switch (right), expulsion of counterions lead to stronger polyelectrolyte interactions and water-rich pockets. (e) Evolution of *G*′ and *G*″ during salt-induced solidification of a hyaluronic acid/chitosan complex coacervate at a fixed frequency. Reproduced from ref. 39, under the terms of the CC-BY-NC 4.0 license. Copyright© 2023 Khoonkari *et al*. Advanced Materials published by Wiley-VCH GmbH.

In several of our studies, ionic strength has served as a primary control parameter to regulate the mechanical state of complex coacervates.^34,35,39,42–46^ Increasing salt concentration screens electrostatic interactions between oppositely charged macromolecules, effectively plasticizing the coacervate and lowering its viscosity and modulus (Fig. 3a and b). This enables injection, extrusion, or printing of the material in a highly deformable state.^40,41^ Subsequent exposure to a lower-salt environment strengthens electrostatic interactions, leading to densification and solidification. In Fig. 3c–e, the underwater adhesive properties of a complex coacervate are illustrated. The initial liquid-like state promotes intimate contact with both the probe and the substrate, while subsequent salt-mediated switching strengthens electrostatic interactions and transforms the coacervate into a cohesive, gel-like adhesive material.

We have exploited this salt-mediated switching to process coacervate-based materials entirely under aqueous conditions. Because coacervates are immiscible with water and possess a low interfacial tension, they do not readily disperse or dissolve upon contact with an aqueous environment. Instead, the coacervate phase remains cohesive and spatially confined at the site of deposition during salt-induced solidification, even under fully submerged conditions. This intrinsic localization enables shaping and subsequent solidification to occur in water without loss of material or dilution, a key requirement for processing and application in wet environments.

**Fig. 4:**
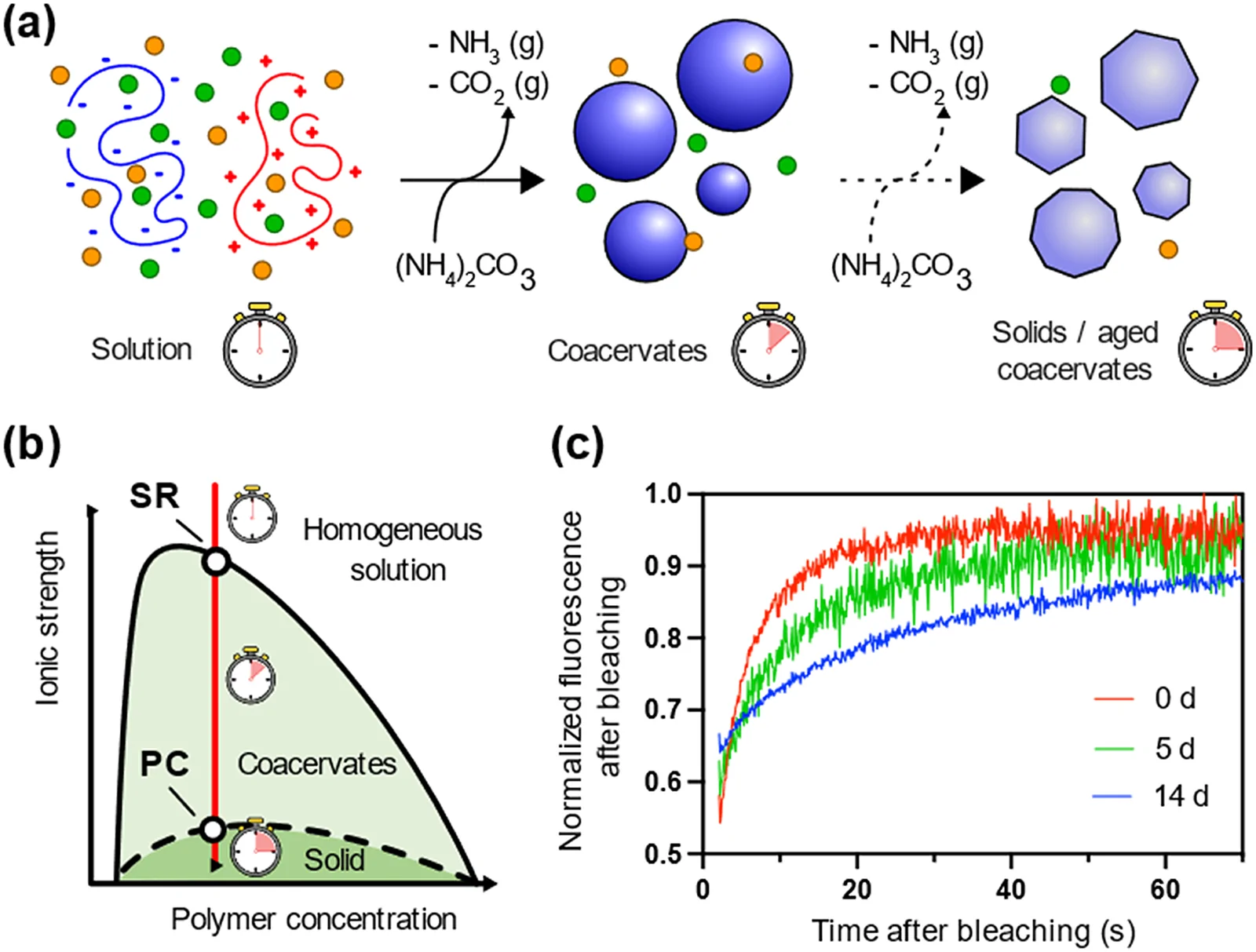
Aging of complex coacervates using a volatile salt. (a) Ammonium carbonate decomposes into NH_3_ and CO_2_, which reduces the ionic strength of the solution and leads to coacervate formation first and rigidification (aging) second. (b) Schematic phase diagram of a system containing two polyelectrolytes, ammonium carbonate, and water, indicating the areas where solution, coacervates, and solid are stable. The salt resistance (SR) and precipitation concentration (PC) are shown, as well as the path that the system takes as ammonium carbonate decomposes and evaporates. (c) Traces corresponding to FRAP experiments performed in coacervates aged for different times. Reproduced with permission from ref. 47. Copyright© 2025 American Chemical Society.

In most salt-switching protocols, changes in ionic strength are achieved by dilution or by submersion of the coacervate into a low-salt bath, leading to diffusive salt exchange with the surrounding medium. More recently, we have explored an alternative route in which the ionic strength of a coacervate suspension is reduced internally rather than externally (Fig. 4).^47^ In this approach, an unstable salt is used as a temporary dopant to form a liquid, plasticized coacervate. Specifically, ammonium carbonate was employed to increase ionic strength during processing. Over time, this salt decomposes into ammonia and carbon dioxide, which escape from the system, resulting in a spontaneous decrease in ionic strength and concomitant rigidification of the coacervate. The opposite reaction could also be achieved, through the *in situ* enzymatic synthesis of ammonium carbonate, which increased the ionic strength of the system and dissolved the coacervates.^48^

This strategy highlights an important extension of salt-mediated switching: salt-dependent changes in coacervate properties can be programmed in time without the need for external solvent exchange or environmental intervention.

### 3.2. pH-Mediated switching: charge regulation and dynamic arrest

In coacervate systems composed of weak polyelectrolytes, pH provides an additional and often orthogonal handle to regulate material behavior. Changes in pH alter the degree of ionization of the polymer backbones and can simultaneously affect polymer solubility and secondary interactions such as hydrogen bonding. pH-mediated switching can be applied locally and dynamically, *e.g.* by means of an evaporating base/acid, allowing solidification to be triggered during or immediately after shaping.^49^ As a result, pH-mediated switching can induce pronounced changes in relaxation dynamics and trigger transitions from liquid-like coacervates to solid-like states.

**Fig. 5:**
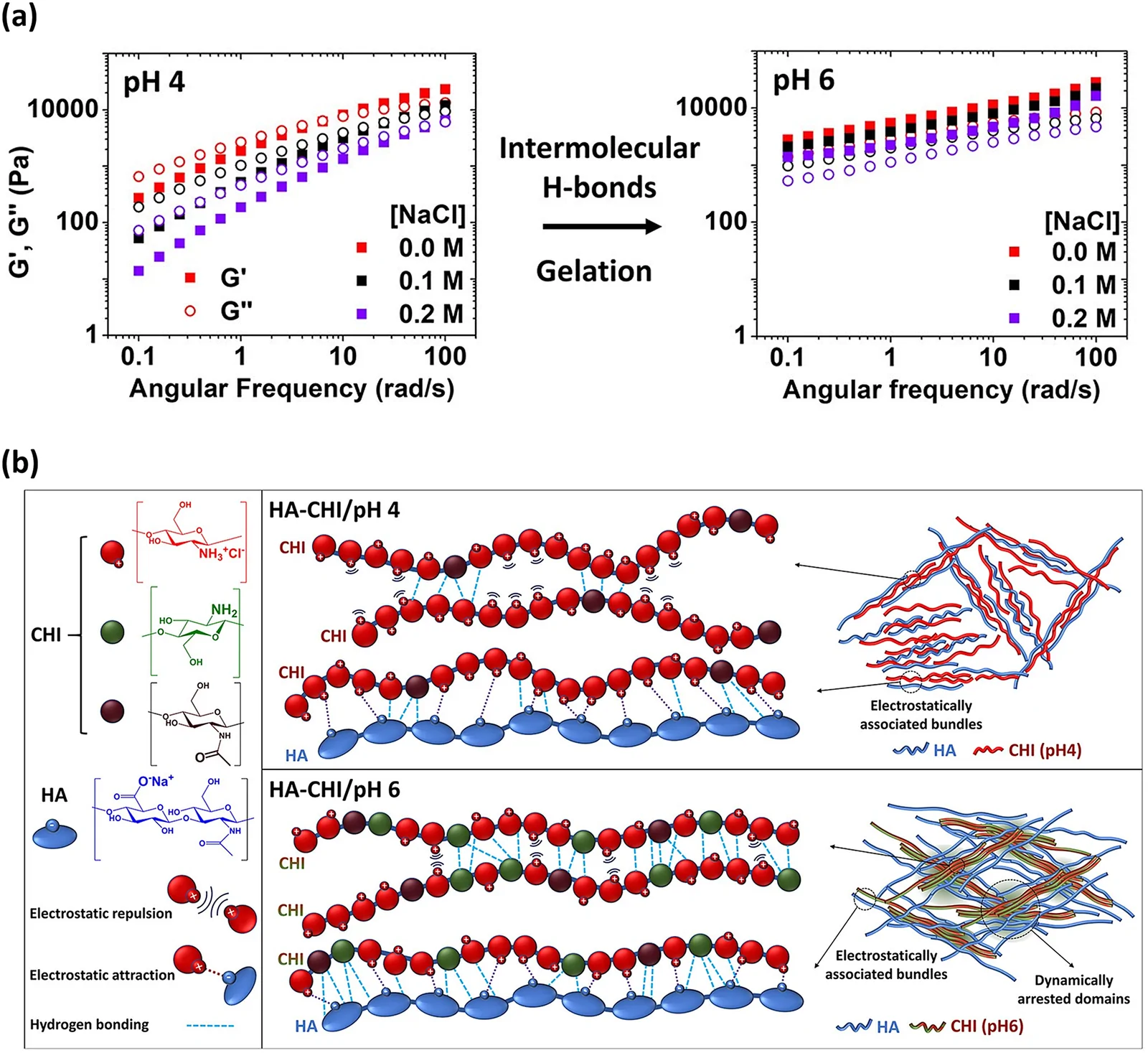
pH-Mediated switching of a complex coacervate. (a) Frequency sweep data for pH 4 and pH 6 at different salt concentrations. (b) Schematic representation of the intermolecular interactions between the polyelectrolyte chains and structure of the complex coacervates at pH 4 and pH 6. Reproduced from ref. 50, under the terms of the CC-BY 4.0 license. Copyright© 2023 Es Sayed *et al*. Published by American Chemical Society.

In our studies, approaching the p*K*_a_ of one of the polyelectrolytes, in this case chitosan, led to the formation of dynamically arrested domains within the coacervate phase through the emergence of hydrogen-bond-rich clusters (Fig. 5).^50^ As shown by the frequency sweep measurements in Fig. 5a, increasing the pH from 4 to 6 strongly alters the viscoelastic response of the coacervate, with a pronounced increase in elasticity and slower stress relaxation, particularly at lower salt concentrations. This behavior originates from the reduced protonation of chitosan near pH 6, where uncharged primary amine groups can readily act as hydrogen bond donors and acceptors. The resulting high density of associative interactions promotes the formation of transient, dynamically arrested domains, schematically illustrated in Fig. 5b. These domains act as transient physical crosslinks that enhance shape retention during processing. We exploited this behavior, for example, to improve print fidelity and structural stability in extrusion-based processing of coacervate inks, without introducing chemical crosslinkers.^39^

### 3.3. Temperature-mediated switching: activation of secondary interactions

Temperature-responsive coacervates provide a complementary mode of environmental switching in which thermal stimuli are used to activate additional interaction pathways rather than to simply induce bulk solidification. In these systems, thermoresponsive polymer segments with a lower critical solution temperature (LCST) are incorporated into the coacervate network.^51,52^ Below the LCST, these segments remain hydrated and weakly interacting, contributing minimally to cohesion while preserving the fluid, processable character of the coacervate. Heating above the LCST selectively triggers collapse of these domains, leading to their association into domains within the polyelectrolyte matrix.

This mechanism allows temperature to act as an orthogonal trigger that reinforces the material without fundamentally altering the electrostatic framework that governs coacervate formation. In contrast to salt- or pH-mediated switching, which directly modulate charge interactions and solubility, temperature primarily controls the engagement of secondary, non-ionic interactions. As a result, thermal activation can be used to increase elasticity, cohesion, or adhesion on demand, while maintaining the overall architecture established during shaping.

In our work on PNIPAM-functionalized complex coacervates, this principle was exploited to introduce thermally addressable reinforcement into underwater adhesives and soft solids. Upon heating above the LCST, PNIPAM-rich domains formed reversible physical crosslinks that enhanced mechanical integrity and resistance to flow, without inducing significant volume changes or chemical reactions.^42–45^ Because the underlying coacervate remains intact, cooling reverses this association, restoring processability and enabling repeated switching cycles.

### 3.4. Modular switching

**Fig. 6:**
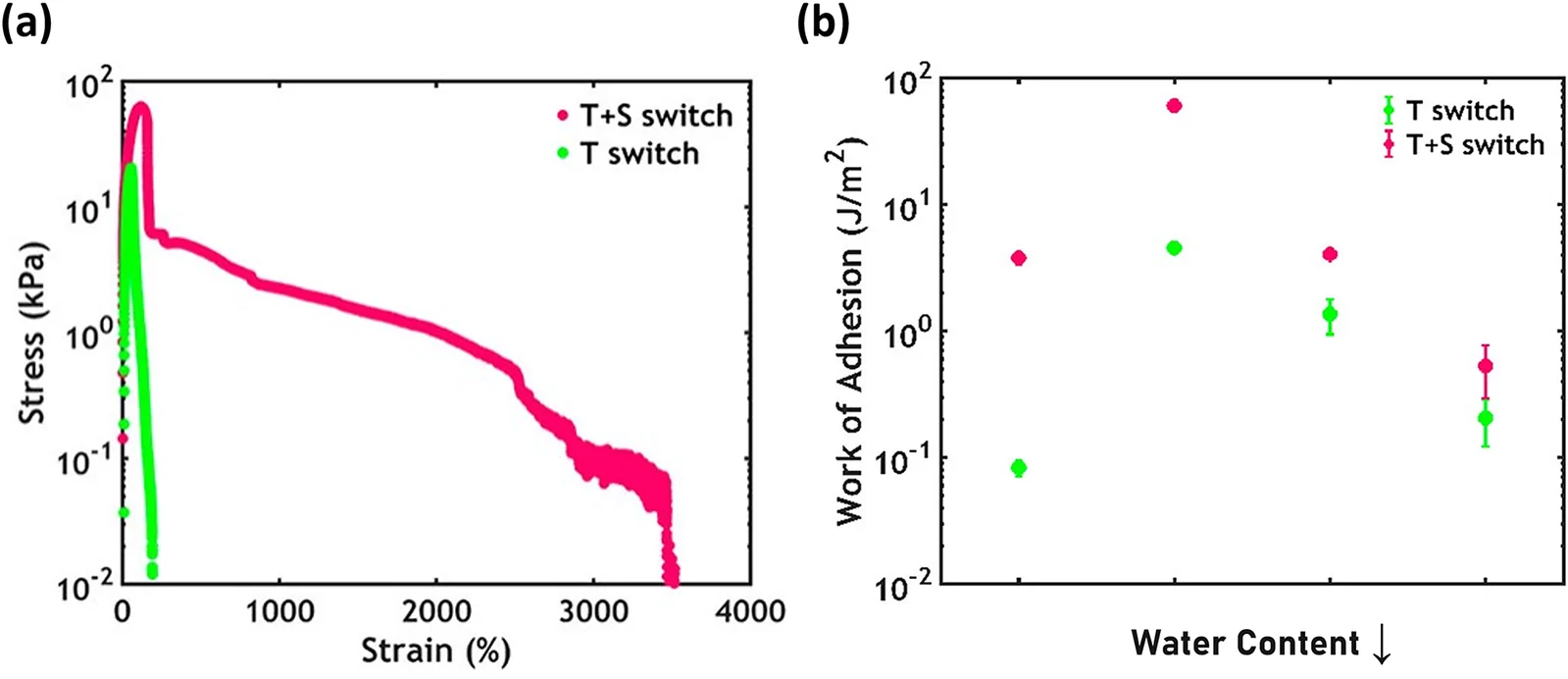
Modular switching of a PNIPAM-functionalized complex coacervate. (a) Underwater adhesive performance after application of a temperature-salt switch (pink) compared to the application of a single temperature switch (green). (b) Work of adhesion *versus* the water content of the complex coacervate *versus* the applied trigger. Adapted from ref. 45, under the terms of the CC-BY-NC-ND license. Copyright© 2020 American Chemical Society.

A key advantage of temperature-mediated switching is that it can be readily combined with salt- and pH-mediated switching, allowing different interaction mechanisms to be activated independently within a single material. In our work on PNIPAM-functionalized complex coacervates, this modularity is clearly illustrated by the fact that mechanical reinforcement can be achieved through multiple, distinct pathways. Changes in ionic strength primarily activate electrostatic interactions within the polyelectrolyte matrix, whereas a temperature increase above the lower critical solution temperature of PNIPAM induces the formation of domains. When applied individually, each trigger leads to a measurable increase in cohesion and adhesion; when applied sequentially or simultaneously, their effects are cumulative, resulting in a substantially enhanced mechanical response (Fig. 6a). By subjecting the complex coacervate to extrusion cycles, the water content can be reduced, further increasing polymer concentration and optimizing the balance between cohesion and adhesion, ultimately leading to significantly improved adhesive performance (Fig. 6b).

This behavior highlights an important characteristic of coacervate-based materials: environmental triggers do not merely switch the material between liquid and solid states, but instead allow access to a spectrum of intermediate states with distinct balances between stiffness, toughness, and energy dissipation. By choosing which trigger to apply, and in which order, the kinetics and extent of solidification can be tuned during or after shaping. That means that, deformation-induced alignment and flow-driven organization can be imposed while the material remains processable and subsequently reinforced by activating additional interactions.

### 3.5. Mechanical deformation-mediated switching

In addition to externally applied triggers, mechanical deformation itself can drive structure formation in coacervate systems.^53^ Seminal work by Linder and co-workers showed that liquid-like protein coacervates can undergo shear- or extensional-flow-induced transitions in which deformation promotes molecular alignment, densification, and the emergence of load-bearing fibrous structures.^54,55^ In these systems, coacervation acts as a necessary intermediate that preassembles proteins into a metastable, deformable state, while elongational flow triggers conformational transitions and hierarchical ordering.

## 4. Coacervate-based material formats and architectures

The processing strategies described above enable coacervates to be shaped into a wide variety of material formats, including fibers, three-dimensional scaffolds, microcapsules, and porous membranes (Table 1).^56^ The combination of high macromolecular concentration, low interfacial tension, and tunable intermolecular interactions allows coacervates to be processed across length scales and geometries that are difficult to access with conventional polymer solutions or melts.

**Table 1:** Summary table of coacervate-based architectures

Architecture	Fabrication Method	Material
Fiber	Wet-spinning	Keratin^35^
		PSS/Keratin^34^
		Alginate/PDADMA^57^
		PSS/PDADMA^58^
		PSS/PEI^59^
	Electrospinning	PSS/PDADMA^62,63^
		PSPMA/PTMAEMA^64^
		Hyaluronic acid/chitosan^65^
	Interfacial complexation	Alginate/chitosan^66^
		Hyaluronic acid/β-lactoglobulin^67^
		Hyaluronic acid/alginate/chitosan^68^
		Chondroitin sulfate/alginate/chitosan^68^
		Glycosaminoglycans/collagen^69^
		PSS/chitosan^70^

Scaffold	Extrusion 3D printing	Hyaluronic acid/chitosan^39^
		Liquid crystalline complex coacervate^46^
		PSS/PDADMA^95^

Microcapsule	Simple coacervation	Gelatin^80,96^
		Zein^97^
		Gliadin^98^
	Complex coacervation	Soy extract/gum arabic^82^
		Pea extract/gum arabic^82^
		Gelatin/gum arabic^83,84,99^
		Collagen/pectin^100^
		Collagen/alginate^100^

Membrane	Salt-induced APS	PSS/PE4VP^90^
		PSS/PDADMA^91,92^
	pH-Induced APS	PSS/PAH^93,94^

### 4.1. Wet-spinning

Fibers and three-dimensional scaffolds represent material formats in which the advantages of coacervates as processing intermediates become particularly apparent. In both cases, shaping relies on the ability to impose flow, deformation, and alignment while the material remains cohesive and highly deformable, followed by solidification that preserves the imposed structure. Our work on both protein- and synthetic polymer-based coacervates illustrates how this principle can be exploited across length scales and material classes.

**Fig. 7:**
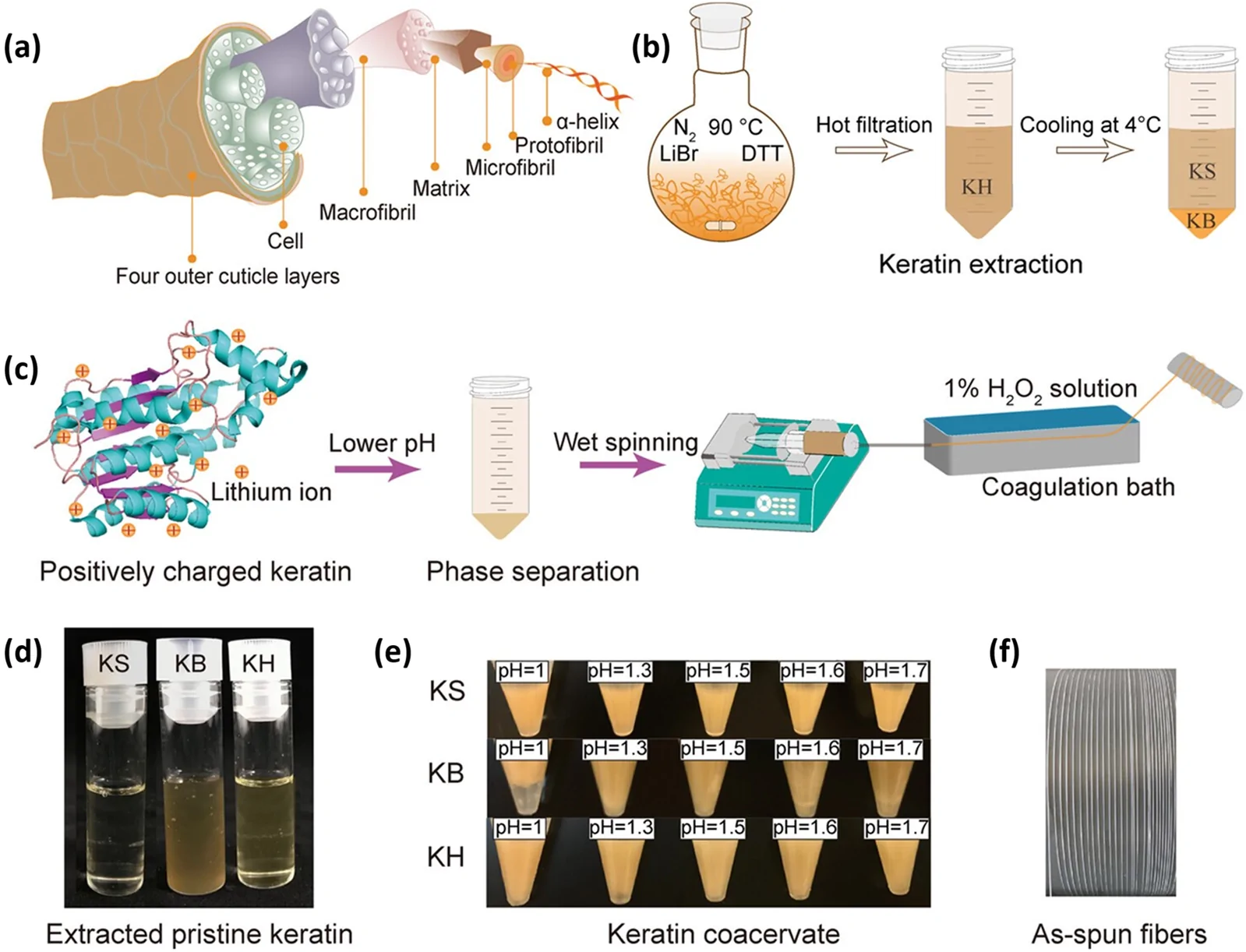
Wet-spinning of keratin coacervates into fibers. (a) Schematic diagram depicting the hierarchical structure of wool fibers. (b) Extraction protocol of keratin from wool: keratin supernatant (KS), keratin bottom (KB), and keratin homogeneous (KH) phases. (c) Schematic representation of keratin coacervate formation followed by wet-spinning processing. (d) Photographs of pristine extracted keratins. (e) Coacervates of KS, KB, and KH at different pH values and (f) as-spun fibers. Reproduced from ref. 35, under the terms of the CC-BY 4.0 license. Copyright© 2023 Sun *et al*. Published by American Chemical Society.

In our studies on keratin, we demonstrated that coacervation provides a viable route to process an otherwise poorly soluble and difficult-to-handle, yet abundantly available, protein into high-performance fibers (Fig. 7).^34,35^ By inducing coacervation through a controlled decrease in pH, extracted keratin was transformed into a dense, liquid-like phase with sufficiently high cohesion to be extruded and wet-spun under mild, aqueous conditions. Importantly, this pH-induced coacervation did not lead to denaturation of the protein. Instead, spectroscopic analysis revealed that a substantial fraction of the native secondary structure was retained during processing. The resulting keratin coacervates could therefore be directly used as spinning dopes, enabling fiber formation without organic solvents, elevated temperatures, or irreversible chemical modification.

Wet spinning of keratin coacervates highlights several hallmarks of coacervate-based fiber processing. Alignment is imposed during extrusion and drawing, while environmental changes lock in the oriented structure. Solidification occurs in a manner that permits stress relaxation during processing, resulting in fibers that combine strength and extensibility. This processing pathway mirrors natural protein fiber formation, where coacervation precedes deformation-induced ordering and solidification, and underscores how processing history, rather than molecular composition alone, governs final material performance.

Wet-spinning was also achieved with synthetic polymers. At sufficiently high salt concentrations, electrostatic interactions are screened and oppositely charged polymers form homogeneous spinning dopes. Upon extrusion into a low-salt or poor-solvent coagulation bath, aqueous phase separation and complexation lead to the formation of solid, porous fibers, with pore size governed by the initial salt content.^57^ Similar processing concepts allow the fabrication of hollow fibers by combining high-salt spinning dopes with bore fluids, followed by phase separation upon salt removal.^58^ When weak polyelectrolytes are employed, pH provides an additional handle to suppress or activate complexation during extrusion, enabling porous and hollow fiber formation *via* pH-induced aqueous phase separation.^59^ Such hollow fiber architectures are particularly attractive for purification and separation technologies, where high surface area, permeability, and mechanical robustness are essential.^60,61^

### 4.2. Electrospinning

Beyond wet spinning, coacervates are also compatible with electrospinning and shear-induced fiber formation. Pioneering studies demonstrated that the high charge density and viscoelastic character of coacervates enable electrospinning even from systems with limited chain entanglement, thereby extending fiber formation to relatively short polymers or oligomeric building blocks.^62–64^ In these systems, electrostatic cohesion within the coacervate effectively replaces conventional entanglement-based stabilization during jet formation.

Subsequent work showed that this approach is applicable to both bio-derived and synthetic polyelectrolyte systems.^62,65^ Moreover, electrospinning from complex coacervates can be combined with selective partitioning of functional molecules, enabling the formation of cargo-loaded fibers through favorable interactions between the cargo and one of the polyelectrolytes.^63^ Together, these studies highlight how coacervation not only enables fully aqueous electrospinning, but also provides intrinsic opportunities for functionalization.

### 4.3. Interfacial complexation

Interfacial polyelectrolyte complexation provides an effective mechanism to stabilize fibers and filaments during processing. When oppositely charged components come into contact at an interface, such as at the boundary between an extruded coacervate and the surrounding solution, complexation can occur locally, leading to the rapid formation of a solid skin. This interfacial reinforcement stabilizes the nascent fiber, enables further drawing, and helps preserve alignment imposed during flow.^66,67^ At smaller length scales, interfacial complexation has also been exploited to fabricate fibers from a wide range of biomaterials by using flow-focusing geometries that precisely control the contact area between two polyelectrolyte solutions.^68,69^ Fine control over the interfacial area and complexation kinetics enables access to more elaborate fiber architectures, including core–shell morphologies.^70^

### 4.4. 3D printing

**Fig. 8:**
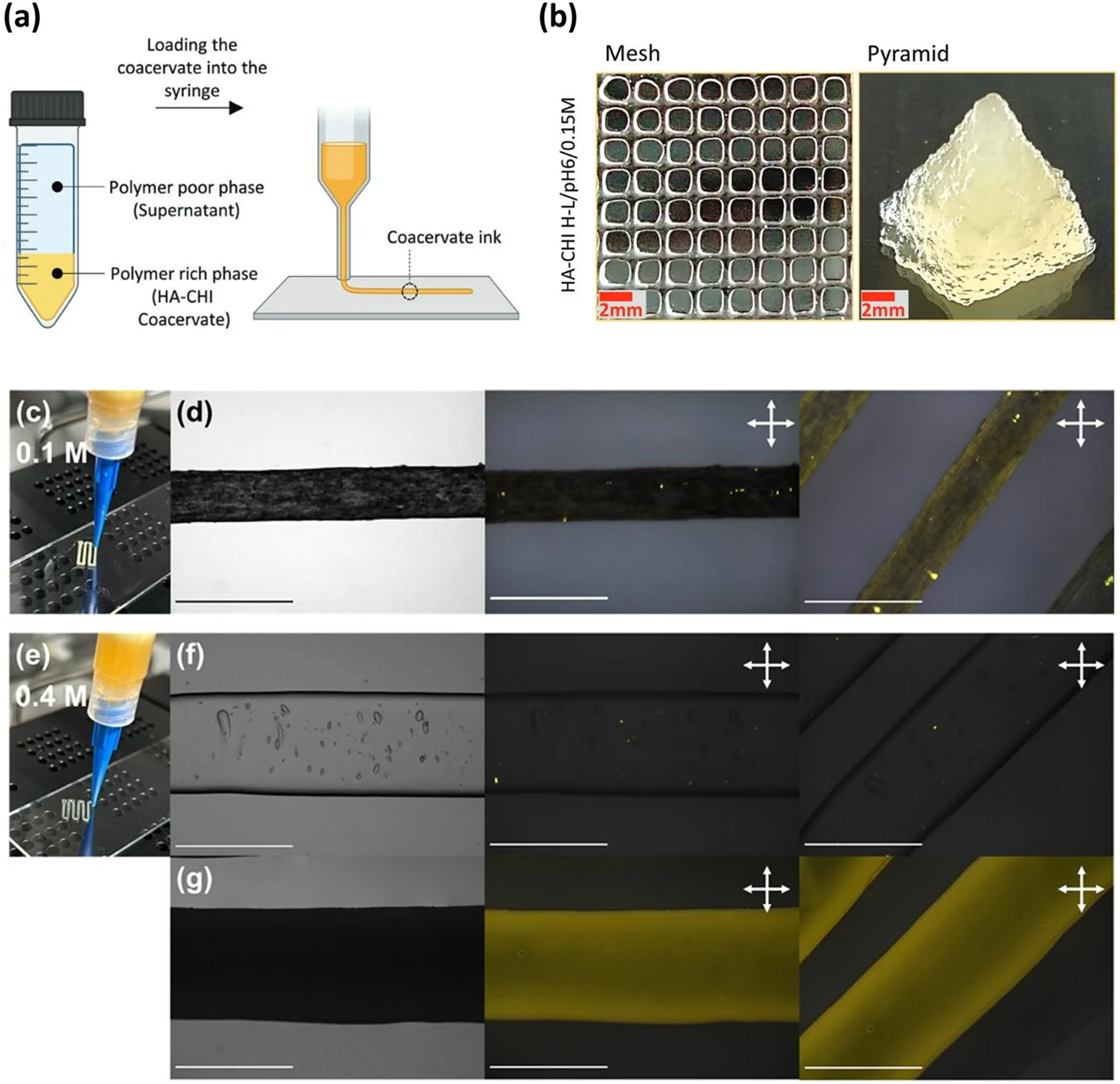
(a) Schematic representation of complex coacervate 3D printing and (b) pictures of a 4-layer square mesh scaffold and a 3D square pyramid structure (15 layers) printed in air. (a) and (b) Reproduced from ref. 39, under the terms of the CC-BY-NC 4.0 license. Copyright© 2023 Khoonkari *et al*. Advanced Materials published by Wiley-VCH GmbH. (c)–(g) 3D printing of liquid crystalline complex coacervates (scale bar 1 mm). Polarized optical microscopy images show birefringence when printed at (d) low salt concentration and after switching from (f) high to (g) low salt concentration. (c)–(g) Reproduced from ref. 46, under the terms of the CC-BY-NC-ND 4.0 license. Copyright© 2026 Liu *et al*. Published by American Chemical Society.

The same principles that enable fiber formation also underpin our work on three-dimensional printing of coacervate-based scaffolds. In this context, coacervates function as viscoelastic inks whose rheological properties can be tuned through composition and environmental conditions. We showed that complex coacervates based on oppositely charged polysaccharides can be formulated to exhibit pronounced shear thinning, allowing smooth extrusion through a nozzle, followed by rapid recovery of solid-like behavior after deposition (Fig. 8a and b). Importantly, the immiscibility of the coacervate phase with water ensures that printed filaments remain localized and stable, even when printing is performed directly into aqueous media.^39^

More recently, we extended this approach to liquid crystalline complex coacervates, demonstrating that ionic strength can be used not only to control printability, but also to regulate internal order and alignment during extrusion (Fig. 8c–g).^46^ By tuning salt concentration, coacervates could be switched between isotropic and liquid crystalline states while maintaining suitable rheology for printing. When printing from liquid crystalline coacervates, the high shear rates inside the nozzle induced pronounced molecular alignment, which was retained after deposition upon solidification (Fig. 8c and d). In contrast, printing from isotropic coacervates (Fig. 8e and f) followed by post-printing salt reduction restored liquid crystallinity without macroscopic alignment (Fig. 8g), highlighting that shear and order must be present simultaneously to generate anisotropic architectures.

Solidification of printed scaffolds can be achieved through environmental switching during or immediately after deposition. Dehydration when printing in air, or changes in ionic strength and pH when printing in solution, strengthen intermolecular interactions without the need for covalent crosslinking or post-printing curing steps. As a result, complex three-dimensional architectures with long-term structural stability and controlled anisotropy can be fabricated entirely under aqueous and biologically relevant conditions. This bioinspired strategy addresses a central challenge in 3D bioprinting, namely the tension between printability, structural integrity, and alignment in wet environments.

### 4.5. Microcapsules and particulate structures

Microencapsulation refers to the compartmentalization of active materials within a protective shell, resulting in microparticles composed of a core containing the active ingredient and a surrounding wall material that provides physical and chemical protection. Coacervation represents one of the earliest and most widely used routes to microencapsulation, with applications spanning food, cosmetics, and pharmaceutical formulations.^71,72^

When a core material is emulsified or dispersed in a polymer solution prior to phase separation, the coacervate-rich phase can adhere to the surface of the core. This adhesion is driven by favorable wetting, enabled by the low interfacial tension of coacervates, and may be reinforced by electrostatic interactions or hydrophobic effects, depending on the system.^73,74^ As a result, the coacervate phase spreads over the core material and forms a shell-like coating.

It should be noted that, when coacervates are used as microcapsules, the coacervate and the core (typically an oil) are not miscible with each other, and the coacervate acts as an intermediate layer that stabilizes an oil-in-water emulsion. This is conceptually different from another strategy, in which coacervate droplets are used to sequester molecules from solutions.^75^ In this second strategy, coacervates are prepared as a suspension of droplets in water, and the compartmentalization of solutes within them is driven by thermodynamically favorable partitioning. The compartmentalization of solutes through partition is a biomimetic strategy, as it reproduces how biomolecular condensates separate and organize biomolecules in the cell.^76^ For this reason, coacervate droplets have been used in several occasions for synthetic biology applications, such as the modulation of enzymatic reactions^77^ or the construction of protocells.^78^ These applications have been extensively reviewed in recent literature,^77–79^ and we will not discuss them in further detail. Instead, we will focus on coacervate microcapsules, as this approach is closer to their other applications as materials, requiring similar processing strategies for stabilization and solidification.

Both simple and complex coacervation have been exploited to encapsulate a wide range of materials, including oils, fragrances, proteins, enzymes (as a water/oil/water emulsion), and other bioactive compounds. In both cases, the common strategy involves (1) preparation of an emulsion of “core” material in water, (2) interfacial coacervation, and (3) crosslinking of the coacervate phase to create a solid capsule. The solidification of the coacervate phase is normally triggered with a chemical crosslinker (*e.g.* glutaraldehyde),^80,81^ but for some coacervates based on proteins, thermal treatment can induce structure changes that can be enough to solidify the shell and further stabilize the emulsion.^82^

**Fig. 9:**
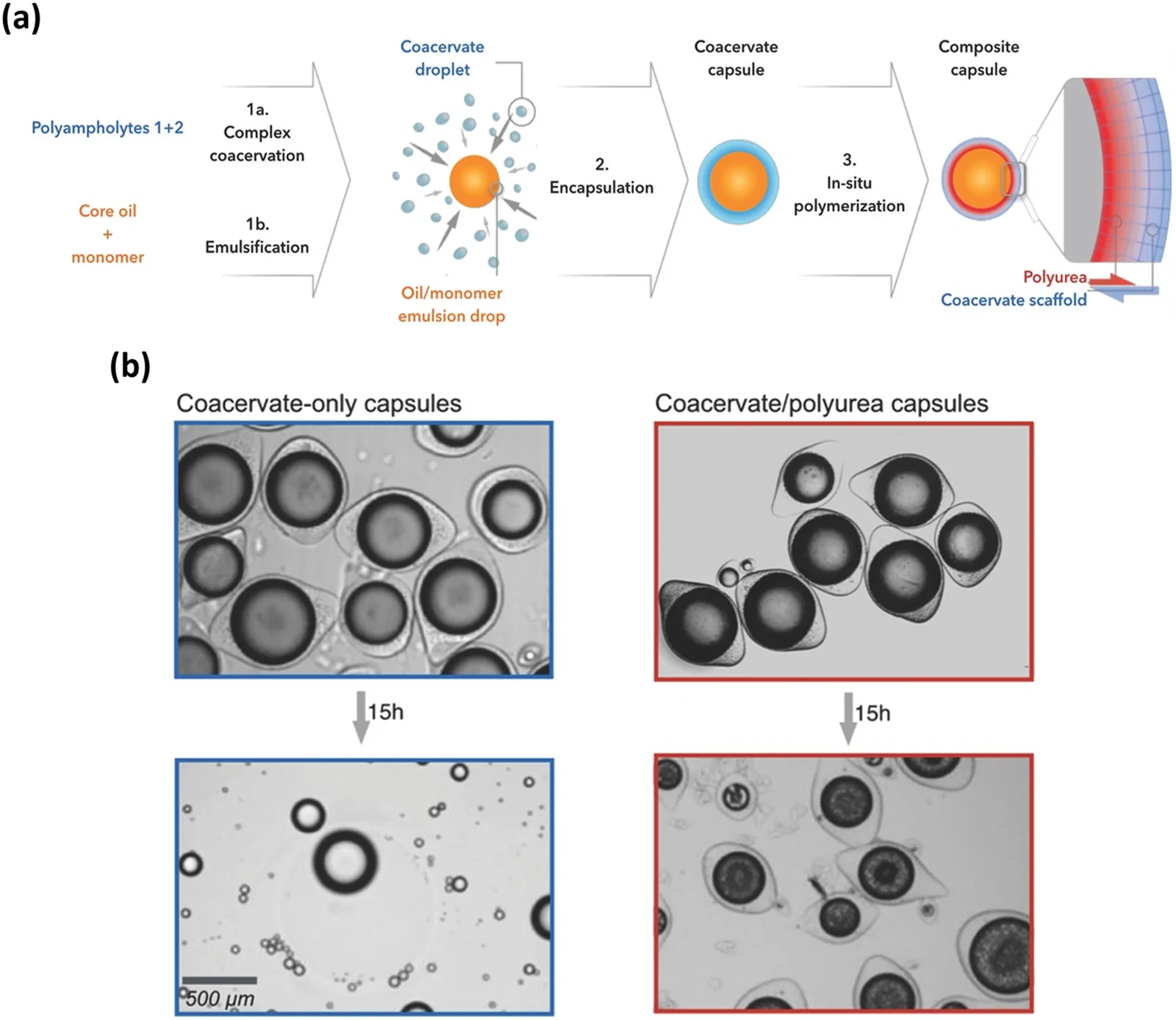
(a) Schematic showing composite core/shell capsule formation resulting in a polyurea/coacervate multilayer structure. (b) Microscopy images of coacervate-only capsules and composite coacervate/polyurea capsules upon exposure to a surfactant solution. Reproduced with permission from ref. 83. Copyright© 2017 Wiley-VCH Verlag GmbH & Co. KGaA, Weinheim.

Beyond conventional oil-in-water capsules, coacervate-based encapsulation strategies have been extended to more complex architectures. Biohybrid core–shell capsules have been developed in which a coacervate shell serves as a scaffold for subsequent reinforcement by inorganic or polymeric barriers, yielding capsules with enhanced mechanical stability and resistance to harsh environments (Fig. 9).^83,84^ Compartmentalization has also been achieved without emulsion templates, for example through the formation of hollow condensates from protein coacervates that undergo controlled reorganization in response to changes in ionic strength, pH, or temperature. These hollow structures possess dense, semipermeable shells capable of encapsulating and releasing active compounds in a regulated manner.^85^

### 4.6. Membranes and porous materials

Synthetic polymer membranes are commonly fabricated by tuning polymer chemistry and processing conditions to control porosity, pore size, and connectivity.^86^ For micro- and ultrafiltration applications, such structural control is essential.^87^ Coacervates and related polyelectrolyte-rich phases provide attractive intermediates for membrane fabrication, particularly in the context of aqueous phase separation (APS). In contrast to conventional membrane production routes that rely on organic solvents, APS enables membrane formation entirely in water using water-soluble polyelectrolytes.^88^ Their charged nature allows high water fluxes, while phase separation provides a direct route to porous structures.^89^ Traditional approaches to polyelectrolyte complex membranes often rely on layer-by-layer deposition, yielding well-defined but typically ultrathin films that are difficult to scale to thicker, mechanically robust membranes.

**Fig. 10:**
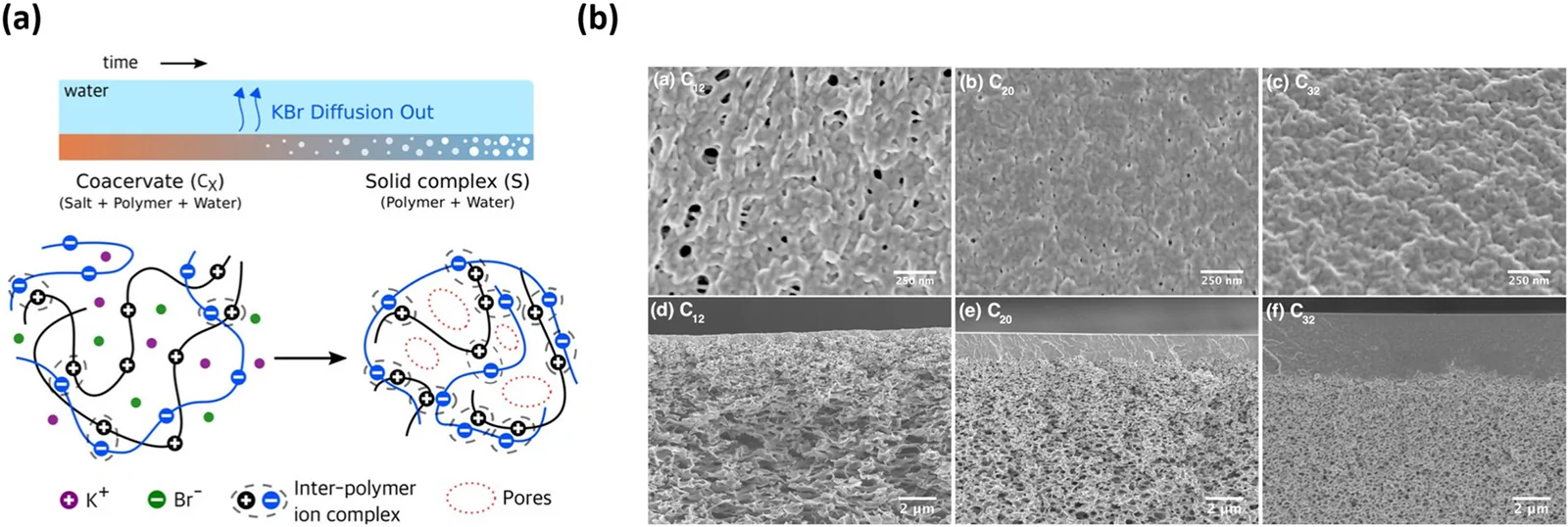
(a) Schematic representation of the salt-mediated water–water phase inversion process of a complex coacervate film to form porous membranes. (b) SEM images of the surface and the corresponding cross sections for membranes cast from initial coacervate compositions with decreasing salt concentrations from left to right. Reproduced with permission from ref. 90. Copyright© 2019 American Chemical Society.

An alternative strategy exploits complex coacervates as membrane precursors. Fluid complex coacervates can be converted into solid porous membranes by salt-mediated switching (Fig. 10).^90^ By varying the salt concentration of the coacervate prior to immersion, the polymer and water content of the precursor phase can be tuned, which directly translates into differences in membrane porosity after phase separation (Fig. 10b). Higher salt contents lead to more open pore structures, while lower salt contents yield denser membranes. The membranes typically exhibit an asymmetric structure, with a dense skin layer atop a porous support, a configuration that combines selectivity with high flux.^60^ A post-processing salt annealing step enabled control over the thickness of the dense skin layer, further illustrating how environmental conditions can be used to tune membrane structure.^91^ In contrast, when membranes are prepared from non-stoichiometric polyelectrolyte mixtures, homogeneous porous structures without a skin layer can be obtained. In this case, excess polycation was proposed to promote spinodal demixing and homogeneous precipitation throughout the film, while increasing polyanion content led to delayed demixing and the formation of a dense top layer.^92^ These examples highlight how membrane morphology is governed not only by composition, but also by the pathway of phase separation.

Beyond salt-induced APS, pH-responsive coacervate systems offer additional routes to porous membrane fabrication. Combining strong polyanions with weak polycations suppresses coacervation at high pH, while lowering the pH induces controlled precipitation into water-insoluble, porous membranes.^93^ Pore size can be tuned by adjusting polymer concentration, molecular weight, and the salinity of the coagulation bath. This approach also enables straightforward incorporation of functional components, for example through the fabrication of biocatalytic membranes by embedding enzymes during pH-induced phase separation.^94^

## 5. Outlook: toward controlled assembly in space and time

Across natural and synthetic systems, coacervates repeatedly emerge as highly effective processing intermediates. Their usefulness does not stem from a single defining property, but from a distinctive combination of physical characteristics that are rarely accessible in conventional polymer processing. Coacervates are dense yet fluid, cohesive yet highly deformable, and capable of responding continuously to changes in their environment. Their high macromolecular concentration, combined with transient, non-covalent interactions, provides connectivity and slow relaxation dynamics even in the absence of classical chain entanglements. As a result, coacervates possess exactly the balance of viscosity and cohesion required for shaping under flow, enabling fiber formation and structuring from relatively short polymers or even oligomeric building blocks—regimes that are inaccessible to dilute polymer solutions or melts.

Beyond their favorable rheology, coacervates provide an environment in which assembly can be guided rather than forced. The dense, liquid-like state allows macromolecules to reorganize, align, and interact under mild conditions, while remaining sufficiently mobile to respond to deformation and external cues. Elongational flow, shear, and local variations in ionic strength, pH, or temperature can induce alignment and conformational rearrangements that are subsequently stabilized as intermolecular interactions are strengthened or additional phases emerge. This coupling between flow, structure formation, and solidification underpins the unique potential of coacervates as processing intermediates.

A central advantage of coacervate-based processing is the ability to decouple where and how a material is shaped from when and how interactions are activated. Unlike conventional processing routes, in which shaping and solidification are often inseparable, coacervates allow structure to be imposed while the material remains deformable, followed by controlled reinforcement at a later stage. Because different interaction modes—electrostatic, hydrophobic, hydrogen bonding, or peptide-based associations—can be addressed independently through environmental parameters, coacervates offer a modular platform in which processing pathways are defined by stimuli rather than composition alone.

The examples discussed in this Feature Article illustrate that a central opportunity lies in moving from abrupt, binary switching toward gradual and spatially resolved control over coacervate properties. In many current approaches, solidification is triggered by rapid changes in environment, such as sudden salt removal or pH jumps, which limit control over internal stress development, heterogeneity, and hierarchical organization. By contrast, gradual changes, implemented through controlled diffusion, internal triggers, or spatial gradients, open routes to steer assembly continuously in space and time. Such strategies more closely resemble biological processing, where materials evolve along defined trajectories rather than undergoing instantaneous transitions.

**Fig. 11:**
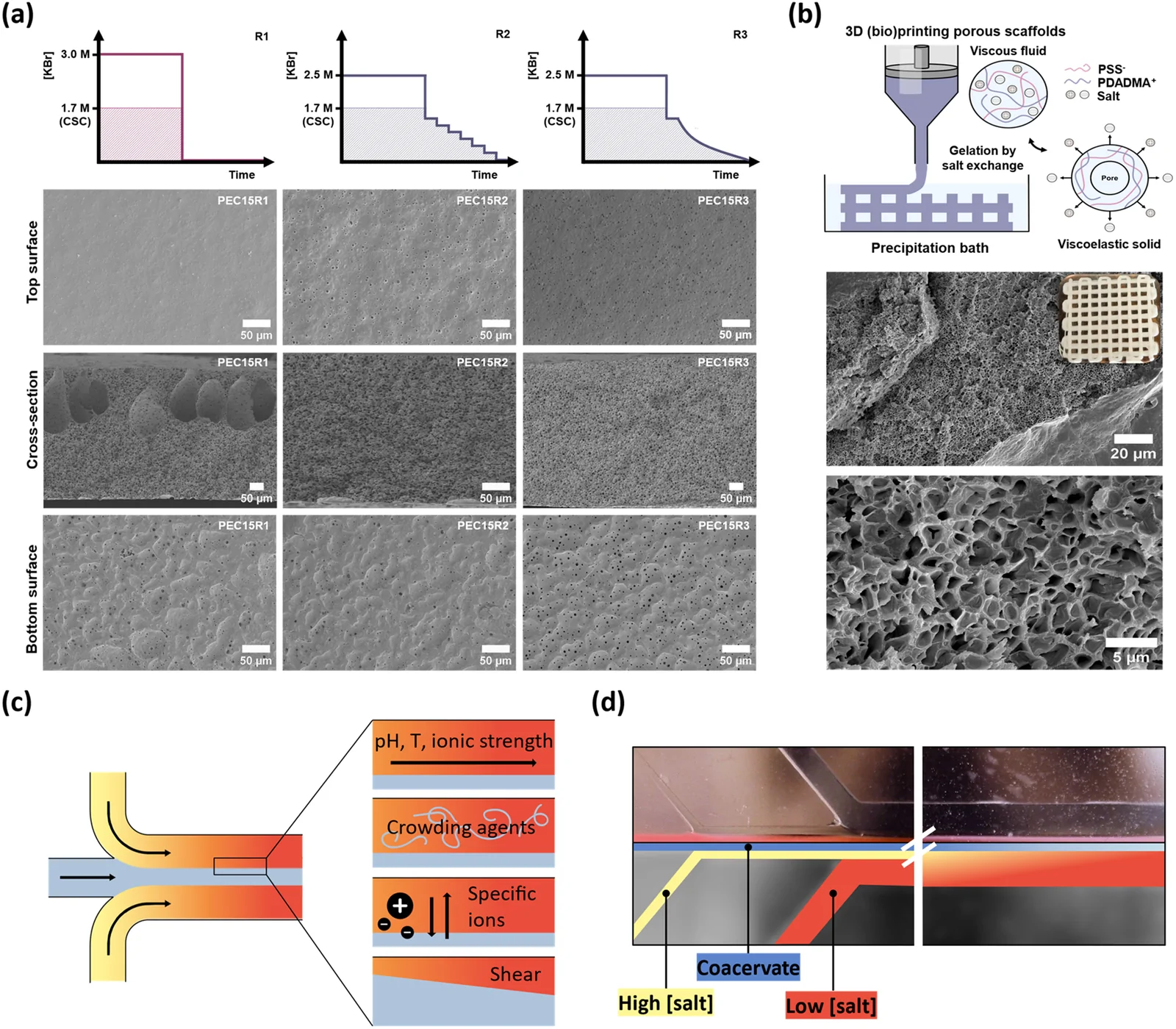
Gradual complex coacervate processing. (a) SEM images of complex coacervate porous membranes produced by instant, step-wise, and gradual salt removal and (b) 3D printing of porous scaffolds by gradual salt removal. Reproduced from ref. 95, under the terms of the CC-BY 4.0 license. Copyright© Mukherjee *et al*. Advanced Functional Materials published by Wiley-VCH GmbH. (c) Microfluidic approaches in fiber spinning to generate directional gradients. (d) Photograph and schematic illustration showing gradual salt-mediated switching of a complex coacervate fiber in a microfluidic chip.

Recent work from our group has begun to move from abrupt to gradual processing of coacervate precursors.^95^ By reducing ionic strength in a controlled and spatially uniform manner, this approach aims to decouple membrane formation from abrupt precipitation and to improve control over pore structure and mechanical integrity (Fig. 11a).

Gradual changes in ionic strength, achieved for example through controlled diffusion or decomposition of unstable salts, provide additional opportunities to steer structure formation and avoid defects associated with rapid coagulation. Such strategies move closer to the gradual processing pathways observed in natural systems.

An emerging insight from both biological and synthetic systems is that time itself plays a decisive role in coacervate processing. In natural silk systems, metastable liquid–liquid phase separation and subsequent aging strongly influence processing outcomes, leading to pronounced path dependence in material properties.^101^ Rather than forming a static precursor, the coacervate evolves over time as molecular rearrangements and secondary interactions slowly develop, meaning that processing history becomes as important as processing conditions. Similar behavior is now being uncovered in synthetic coacervate systems. Recent work from our group has shown that peptide-containing coacervates can undergo salt-induced aging, during which secondary structure formation and crosslinking proceed gradually after phase separation, leading to time-dependent changes in mechanics and processability. In these systems, electrostatic interactions define the initial coacervate state, while slower peptide-mediated interactions progressively reinforce the material, introducing a temporal hierarchy of interactions.^102^

These observations point toward a broader vision in which coacervates are not treated as equilibrium phases, but as evolving materials whose properties depend on controlled trajectories through composition, environment, and time. By deliberately exploiting gradual changes through diffusion-limited salt exchange, decomposable salts, slow pH shifts, or temperature ramps, it becomes possible to steer assembly in both space and time. Such approaches move beyond binary switching and open routes to control alignment, porosity, phase separation, and mechanical gradients during shaping rather than after it.

Looking forward, we envision coacervate-based processing as a platform for programmed materials assembly, in which structure and function are encoded in environmental pathways. Combining multiple switching mechanisms within a single system enables distinct interaction modes to be activated sequentially or locally, allowing selective locking-in of flow-induced organization, and coupling of deformation directly to molecular ordering. Additive manufacturing (Fig. 11b) and microfluidic processing (Fig. 11c and d) are particularly promising tools to realize this level of control, as they offer precise control over gradients, residence times, and deformation histories.

Ultimately, coacervates invite a shift in perspective: from materials defined primarily by composition to materials defined by processing history. By embracing gradual, stimulus-driven control over assembly in space and time, and by recognizing time as an active design parameter, coacervates offer a powerful route toward bioinspired materials with unprecedented levels of structural and functional control.

## Author contributions

The manuscript was written through contributions of all authors. All authors have given approval to the final version of the manuscript.

## Conflicts of interest

There are no conflicts to declare.

## Data Availability

No primary research results, software or code have been included and no new data were generated or analyzed as part of this review.
